# The Effect of Particle Size on Thermal Conduction in Granular Mixtures

**DOI:** 10.3390/ma8073975

**Published:** 2015-07-02

**Authors:** Junghwoon Lee, Tae Sup Yun, Sung-Uk Choi

**Affiliations:** 1 Department of Civil and Environmental Engineering, Georgia Institute of Technology, North Ave NW, Atlanta, GA 30332, USA; E-Mail: junghwoon@gatech.edu; 2 Department of Civil and Environmental Engineering, Yonsei University, 50 Yonsei-ro, Seodaemun-gu, Seoul 120-749, Korea; E-Mail: schoi@yonsei.ac.kr

**Keywords:** thermal conductivity, rubber mixture, size ratio, discrete element method, network model

## Abstract

Shredded rubber tire is a geomaterial that is potentially useful in environmental and engineering projects. Here, we study the effect of particle size ratio on the thermal conductivity of granular mixtures containing rubber tire particles. Glass beads were mixed at various volume fractions with rubber particles of varying size. The 3D network model analysis using synthetic packed assemblies was used to determine the dominant factors influencing the thermal conduction of the mixtures. Results present that mixtures with varying size ratios exhibit different nonlinear evolutions of thermal conductivity values with mixture fractions. In particular, mixtures with large insulating materials (e.g., rubber particles) have higher thermal conduction that those with small ones. This is because the larger insulating particles allow better interconnectivity among the conductive particles, thereby avoiding the interruption of the thermal conduction of the conductive particles. Similar tests conducted with natural sand corroborate the significant effect of the relative size of the insulating particles. The 3D network model identifies the heterogeneity of local and effective thermal conductivity and the influence of connectivity among conductive particles. A supplementary examination of electrical conductivity highlights the significance of local and long-range connectivity on conduction paths in granular mixtures.

## 1. Introduction

Varying the properties of the constituents of granular mixtures has been widely used to modulate the geomechanical and physical behaviors of mixtures containing, for example, crumb rubber, shredded tire and fly and bottom ash. It is well known that the volumetric fraction of a mixture and its boundary conditions determine its physico-mechanical properties. In particular, rubber particles with low density, stiffness and conductivity can be mixed with soil to decrease its effective unit weight, to improve its thermal insulation and to modulate strain-dependent stiffness and other properties of interest, including the maximum shear stiffness damping, time-dependent settlement, thermal conductivity and shear strength [1,2,3,4,5,6]. Damping and creep strain, shear stiffness and thermal resistivity have been found to increase with an increasing fraction of crumb rubber. Producing granular mixtures that contain rubber particles therefore seems an attractive method to recycle a waste material and to obtain useful engineering properties. The thermal conduction of mixtures is strongly affected by the presence of thermally-insulating materials, because of the inhomogeneous distribution of such materials at the particle-scale and the threshold value that delineates the non-conductive regime [7]. This observation can be analogously extended to electrical conduction to explore the formation of percolation. For electrical conduction, percolation in a granular mixture depends mainly on the properties of the first nearest neighboring particles, whose inter-connectivity changes with the fraction of conductive granules and the application of stress.

The thermal conductivity of soils must be considered in the design of backfill materials for pipelines and in improving the ground susceptibility to freezing. Rubber particles constitute a promising additive to enhance thermal insulation because of their low thermal conductivity [8]. Increasing the fraction of less conductive rubber particles decreases the thermal conductivity of the rubber-soil mixture, and their effect on thermal conductivity has been quantitatively estimated in physical investigations [7]. The dominant factors considered in such investigations are commonly limited to the volumetric fraction, size of each constituent and the presence of pore fluid. Soil particles are more thermally conductive than other soil constituents (e.g., pore fluid and air); therefore, the inter-particle contacts provide the main heat transfer path in a granular mixture (other factors, such as mineralogy, particle size, water content and applied pressure also affect conductivity) [9]. The inter-particle conditions (e.g., coordination number, overlapped contact area and contact quality) are critical to the effectiveness of heat transfer in granular materials, and thus, the relative sizes of the particles of differing conductivity should greatly influence the estimation of the effective thermal conductivity of rubber-soil mixtures. In other words, the interconnectivity among the highly-conductive particles (e.g., soil particles) varies depending on how the less conductive particles are spatially configured in the granular assembly.

The present paper reports experimental measurements of the thermal conductivity of granular mixtures. Particular attention is paid to the relative sizes of the rubber and soil particles in the granular mixtures and to the ensuing engineering implications. The effects of varying the volumetric fraction and the relative particle size of each constituent are tested in conjunction with numerical analysis. The transient plane source method is adopted for thermal measurement, and the evolution of thermal conduction is estimated using the network model of synthetic granular mixtures performed with the discrete element method (DEM). The thermal conductivity of the granular mixture is determined not only by the fraction of each constituent but also, more importantly, by the particle size ratio.

## 2. Materials and Methods

### 2.1. Materials

Granular mixtures of rubber particles and glass beads were prepared with different particle size ratios [10]. The size ratio $R_{D}$ $\left(=D_{\mathrm{insulating}}/D_{\mathrm{conductive}}\right)$ is defined as the ratio of the diameter of the insulating rubber particles to the diameter of the conductive glass beads. Mixtures with three different size ratios were prepared ($R_{D}$ ∼ 1, 4 and 0.25) using smooth, spherical, soda lime glass beads ($\mathrm{CaMgO}-{\mathrm{Na}}_{2}O-{\mathrm{SiO}}_{2}$, a mean diameter of 0.212∼0.3 mm) and shredded tire chips sieved to the designated size (see Table 1). Based on their specific gravity, the two components were weighed and stirred to reach designated volumetric fractions of glass beads relative to the combined volume of the glass beads and the rubber ($F_{GB}$ = 0 (rubber only) to 100% (beads only) at 20 percentage point increments). The mixtures were sufficiently mixed in a dry condition to achieve homogeneity. The thermal conductivity of the glass beads ($k_{GB}∼1.1$ W/mK) was about five times that of the rubber particles ($k_{R}∼0.25$ W/mK). Each mixture was placed by air-pluviation into a cylindrical cell (diameter 30 mm, height 30 mm). The initial porosity of specimens increases with increasing rubber fraction as summarized in Table 2. This was due to the different compressibilities of and the seating effect driven by self-weight. Confining stress was however not applied, because the rubber particles are very sensitive to compression, and such confining stress may otherwise have altered the inter-particle contact condition and the resultant thermal conductivity values. Overall, the thermal conductivities of 21 mixtures of varying size ratios and mixture fractions were measured, and each case was repeated three times.

**Table 1:** Cases in glass bead-rubber mixture experiments.

Case	Particle Diameter [mm]		$R_{d}$
	Glass Bead	Rubber	
Case 1	0.212∼0.3	0.25∼0.3	∼1
Case 2	0.212∼0.3	0.85∼1.13	∼4
Case 3	0.212∼0.3	∼0.112	∼0.25

#### Method

**Figure 1:**
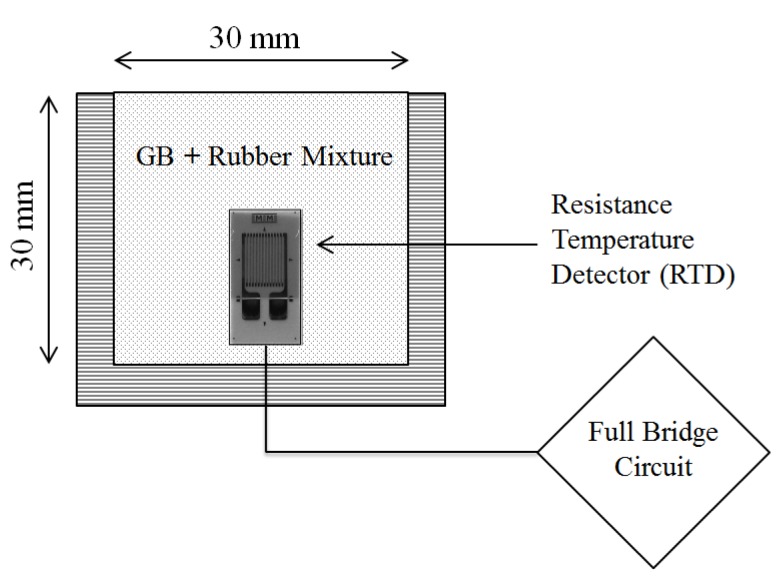
Experimental configuration for measuring thermal conductivity of granular mixtures. The transient plane source method embedded within the specimen is implemented to measure thermal conductivity.

The transient plane source method was used to measure thermal conductivity [9,11]. A pair of resistance temperature detectors (RTD-ETG 50B/W, Vishay) with a known resistance of 50 Ω served as both the heat source and temperature detector. Two 50 Ω resistors were connected to make a full-bridge circuit. The differential voltage across the circuit began changing once the applied constant VDCgenerated the plane heat, and its incremental variation depended on the thermal conductivity of the surrounding materials. The voltage and applied currents were monitored every 0.1 s for 30 s to compute the temperature variation and the applied thermal energy. Thereafter, minimizing the differences between the measured temperature change values and the theoretical values allowed the effective thermal conductivity of the mixture to be computed. The sensor was installed at the center of the specimen, as shown in Figure 1. For each specimen, the measurement was repeated three times at intervals of 5 min. Details of the thermal measurement and its validation can be found in [11].

**Table 2:** Volumetric fraction and porosity of glass bead-rubber mixture.

**Vol. Fraction, $F_{GB}$ (%)**	Porosity		
	Case 1	Case 2	Case 3
0	0.65	0.63	0.59
10	0.65	0.58	0.55
22	0.62	0.61	0.59
39	0.55	0.55	0.48
63	0.51	0.48	0.44
80	0.38	0.40	0.39
100	0.40	0.40	0.40

## 3. Numerical Simulation

The thermal network model from our previous numerical study [12] was used to estimate the effective thermal conductivity of the mixtures using 3D virtual specimens generated by the discrete element code $PFC^{3D}$.

### 3.1. Generation of Packing

**Figure 2:**
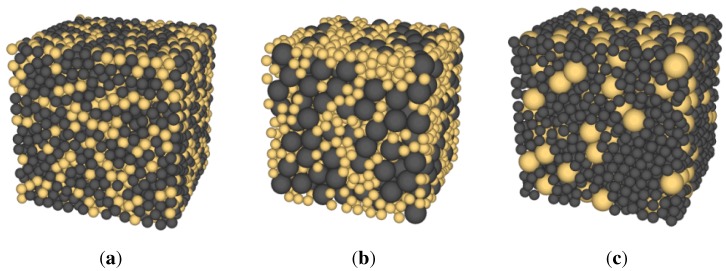
Particle assemblies for glass bead (GB) and rubber (R) mixture ($F_{GB}$ = 40%) where the yellow color denotes glass beads and a dark color indicates rubber particles (**a**) Case 1: $R_{D}=D_{R}/D_{GB}∼1$; (**b**) Case 2: $R_{D}=D_{R}/D_{GB}∼4$; (**c**) Case 3: $R_{D}=\;D_{R}/D_{GB}∼0.25.$

The discrete element method (DEM) was implemented to generate a packed assembly that mimics a distributed and heterogeneous mixture. The numbers of glass beads and rubber particles were first determined based on the given volumetric fraction and size ratios (Table 1). Particle seeds were then randomly placed within the smooth (wall friction = 0) and rigid cubic container; subsequently forming the packed assembly of the mixture by using radius expansion method. The size of the container varied depending on the maximum number of particles allowed in the $PFC^{3D}$. Each assembly therefore had more than 3000 particles, so as to satisfy the representativeness of the microscopic behavior of a granular material [13]. The particles were mobilized with the material properties summarized in Table 3 using the Hertz–Mindlin model. Unlike with the experiments, an isotropic confinement of 10 kPa was applied to the assembly to prevent floating particles. Sufficient time was allowed for the system to reach an equilibrium state with a safety factor of 0.17 [14,15] and to prepare the stabilized assembly that served as the basic domain for the thermal analysis. Figure 2 illustrates the numerically-generated packed assemblies by the DEM.

**Table 3:** Material properties and model parameter for the DEM simulation and network model.

	Values	Rubber Particle	Glass Bead	Chrome Ball
DEM	Specific gravity	1.05	2.51	7.81
	Shear modulus (MPa)	$2.9\times 10^{1}$	$2.9\times 10^{4}$	$8\times 10^{4}$
	Poisson’s ratio	0.5	0.31	0.31
	Friction coefficient	0.5	0.31	0.31
Network model	Thermal conductivity (W/mK)	0.25	1.124	-
	Electrical resistivity [nΩ·m]	-	$10^{19}$ to $10^{23}$	219

#### Network Model

**Figure 3:**
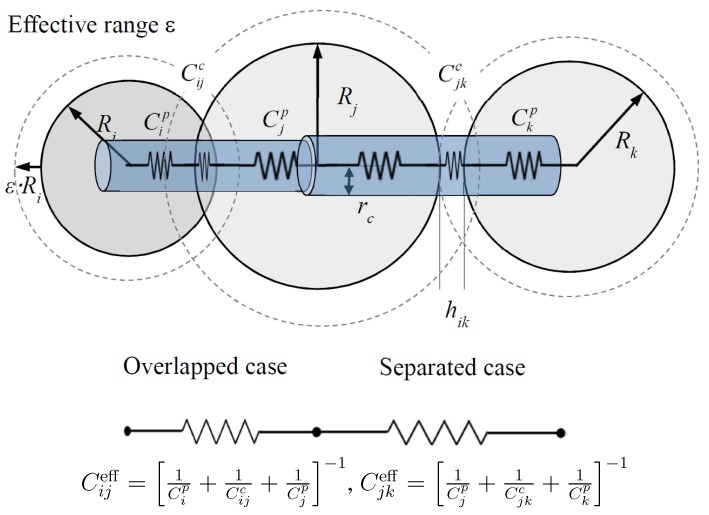
Illustration of generalization of thermal conductance between particles in the 3D random network model. The particle iis overlapped with particle j. Though particle k is separated from particle j, their distance is closer than the effective zone, which is determined by cutoff range parameter *ε*.

The thermal network model used in this study was based on the construction of a 3D web of a thermal conductance network that depends on inter-particle contact properties, such as particle size, overlapped area or separation distance between adjacent particles, material conductivity and the effective zone of conduction. Its applicability to granular materials has been previously validated with respect to representativeness, porosity and loading effects [12]. As the radii and coordinates of the particles in the assembly were known from the virtual specimens through DEM simulation, the conductance of each particle and contact conductance can be computed by following the theoretical derivation and its numerical implementation [12,16]. Figure 3 illustrates the schematic concept of the 3D web, which is based on inter-particle connections and the contact properties used in this simulation. Each particle contact and each particle itself can be represented by a series of thermal conductance springs, which, in turn, symbolizes the effective thermal spring between particles. There are two main model parameters that control the calculation: the estimate of the fraction of the mean radius of particle curvature *χ* and the cutoff range parameter defining the effective zone *ε*. The model then calculates the effective thermal conductivity of the mixture based on the 3D web of thermal conductance springs under the constant temperature boundary conditions imposed at both the top and the bottom. For thermal conduction (e.g., soft percolation), heat can propagate through both overlapped particles and particles that are slightly separated. Therefore, the cutoff range parameter *ε* defines the effective zone within which heat can be transferred between materials via the pore space. Constant temperatures of 5 ${}^{\circ }$C and 1 ${}^{\circ }$C were assigned to the top and bottom, respectively, and the temperature of each particle was iteratively computed until the temperature variation at consecutive steps became zero. The conductivity values at 50 different cross-sectional planes along the height were obtained based on Fick’s law, and the harmonic mean value was computed to obtain the effective thermal conductivity. The detailed implementation and its validation with experimental results can be found in [12]. The model parameters were parametrically determined to match the experimental results in this study.

## 4. Results

### 4.1. Effective Thermal Conductivity

**Figure 4:**
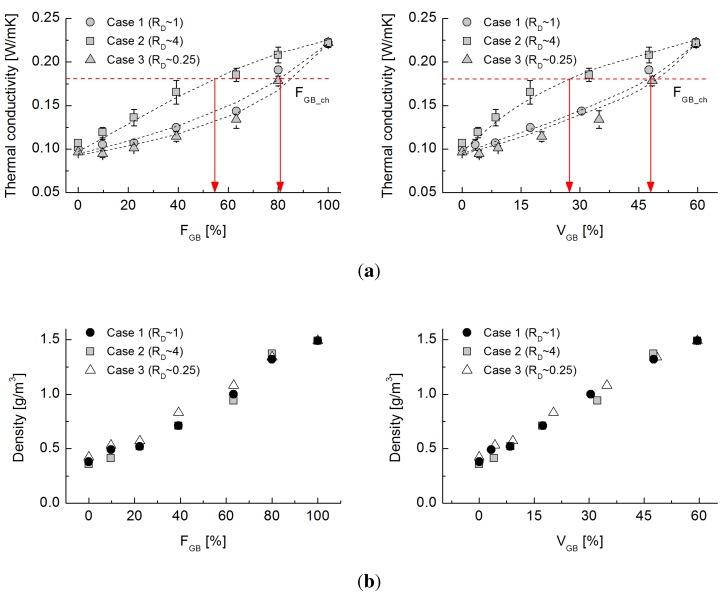
(**a**) Evolution of thermal conductivity values with varying volumetric fractions of glass beads for Cases 1 to 3; (**b**) density values with varying volumetric fractions of glass beads for Cases 1 to 3.

The thermal conductivity values of the three glass bead-rubber mixtures are plotted with respect to $F_{GB}$ and also to the volumetric fraction of glass beads with respect to the total specimen volume, $V_{GB}$, in Figure 4. For example, an $F_{GB}$ of 20% indicates that 20% of the solid volume consists of glass beads and 80% of the volume is rubber particles; if the porosity of the mixture is 0.4, then $V_{GB}$ is 12% (*i.e.*, $V_{GB}=\left(1-n\right)\cdot F_{GB}$). As the thermal conductivity of the granular mixture should be influenced by the mixing ratio, as well as the porosity, and as the porosity of the experimentally-tested specimens varies for each mixture, samples of equal $F_{GB}$ that have different porosities do not have equal volumes of glass beads (*i.e.*, they do not have equal $V_{GB}$). Hence, defining $V_{GB}$ helps to eliminate the effect of porosity by directly indicating the volumetric portion of glass beads in the whole volume.

The glass beads alone ($F_{GB}=100\%$) exhibit a thermal conductivity of 0.22 W/mK, whereas the rubber particles ($F_{GB}=0$) have a value of 0.1 W/mK, regardless of their size. This indicates that thermal resistance at the inter-particle contacts prevails in the granular state and that nominal changes in particle size have less effect [8]. Regardless of the particle size ratio, the effective thermal conductivity increases with the increasing fraction of glass beads, because the conduction is facilitated through their high conductivity. However, the trend in Case 2, in which the less conductive rubber particles are four-times larger than the glass beads ($R_{D}∼4$), runs above Cases 1 and 3 through the entire range of $F_{GB}$, thereby exhibiting quasi-convex evolution. The mixtures in Case 2 have large rubber particles as anomalous forms interspersed among a well-connected network of conductive glass beads that provides highly conductive heat paths. However, the smaller rubber particles in Case 3 ($R_{D}∼0.25$) are more effective at interrupting the conduction chain compared with Case 2. The conduction chains made by the different particle size ratios result in the observed differences of thermal conductivity among the tested cases. In Figure 4a, the dotted lines are trend lines, and the variation derives from repeated testing (e.g., nine measurements for each). Case 2 has the largest variation, which implies that a mixture with larger, less conductive particles is more easily influenced by the spatial configuration of the particles compared with a mixture with smaller insulating particles.

The characteristic fraction $F_{GB\_ch}$ is defined as the glass bead fraction that corresponds to the value of $(k_{max}-k_{min})/e$. The characteristic fraction for the data shown in Figure 4a is 55% for Case 2 and 82% for Cases 1 and 3. In other words, the constituent fractions of the mixtures can vary greatly and yet achieve similar reductions in thermal conductivity. This observation confirms that the thermal conductivity of the granular mixture is determined not only by the fraction of each constituent, but also, more importantly, by the particle size ratio. It enables a design to satisfy both unit weight and thermal conductivity. The density of each mixture plotted in Figure 4b indicates that the values are generally consistent regardless of the particle size ratio, showing quasi-linear increments with an increasing proportion of glass beads. Overall, these findings demonstrate that the size ratio, in addition to the mixture density, should be specified to modulate the thermal conductivity of a mixture.

### 4.2. Network Model

**Figure 5:**
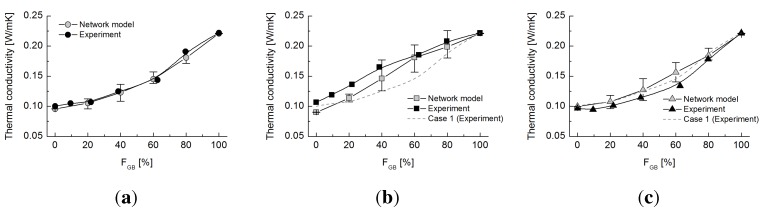
Thermal conductivity obtained by experiments and network model for Cases 1 to 3. (**a**) Case 1; (**b**) Case 2; (**c**) Case 3.

The thermal conductivities estimated by the 3D network model are superimposed on the experimental results in Figure 5. The dominant model parameters, that is the effective zone parameter *ε* and the particle curvature parameter *χ*, were determined as follows. Adequate values of *ε* and *χ* were first determined to match the values for $F_{GB}$ = 0 and 100%. A single value of *ε* was selected, whereas different values of *χ* were manipulated based on the type of contact between materials (e.g., glass bead-glass bead, rubber-rubber). These values were then systematically changed until they fitted the experimental results. Finally, the parameter *ε* was set at a value of 0.50, whereas parameter *χ* was established at a value of 0.85 for glass bead to glass bead contact, 0.75 for rubber to rubber contact and 0.50 for glass bead to rubber contact. As the curvature parameter *χ* modulates the thermal conductance between adjacent particles, it is reasonable to determine that the highest value of this parameter is for glass bead particle contacts (which provide the main heat-transfer path), whereas glass-rubber contacts have the lowest value, as thermal transfer between these two different materials is ineffective. The network model appears reasonable, as it captures the overall tendency of thermal conductivity with the varying fraction of glass beads and varying particle size ratio. It is noted that both values were set to 0.50 in our previous study of the thermal network model for dry sand specimens during model validation [12].

Appropriately-estimated values of *ε* and *χ* capture the effect of particle size ratio on thermal conductivity despite the porosity differences between the numerically-generated particle assemblies and the experimental specimens. Neither of these parameters, *ε* and *χ*, is able to provide deterministic insights into this observation. However, from the physically-suitable range of model parameters in our numerical simulation, we conclude that the size ratio is the dominant influence on thermal conductivity and that porosity has less influence.

### 4.3. Dominant Factors Influencing Thermal Conduction

**Figure 6:**
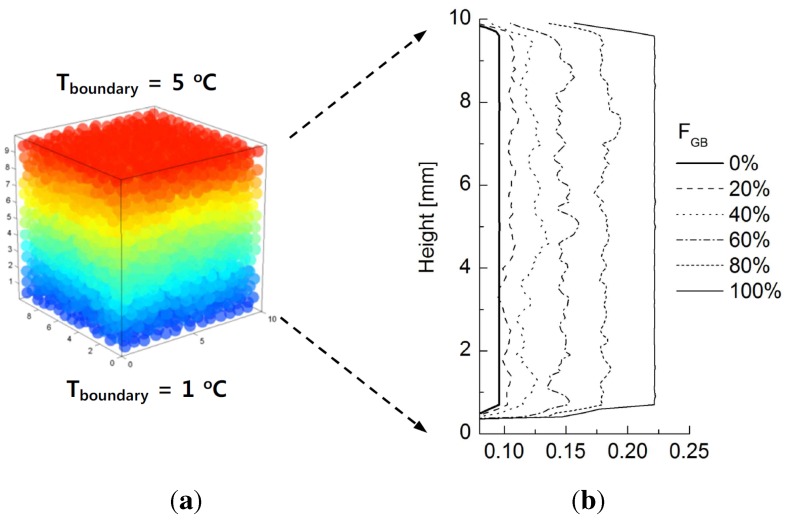
(**a**) Temperature distribution with respect to the height for $F_{GB}$ = 60% in Case 1 at steady-state condition; (**b**) profiles of estimated thermal conductivity values with respect to the height for different fraction of glass beads.

Figure Figure 6a illustrates the temperature configuration of particles computed by the network model for $F_{GB}$ = 60% in Case 1. As mentioned in the previous section, the thermal conductivity at any selected cross-sectional plane can be calculated so that its profile along the height of the sample can be made as shown in Figure 6b. The pure specimens ($F_{GB}$ = 0 and 100%) exhibit constant conductivity values. However, the mixtures show variations in conductivity along their height, owing to their heterogeneity and random distribution. The average values of thermal conductivity are the same as those plotted in Figure 5b. This observation suggests that heat does not uniformly propagate throughout the mixture, because the conduction across inter-particle contacts differs depending on the composition of the particles. Furthermore, the thermal network model allows the thermal conductivity of the different mixtures to be accurately estimated.

**Figure 7:**
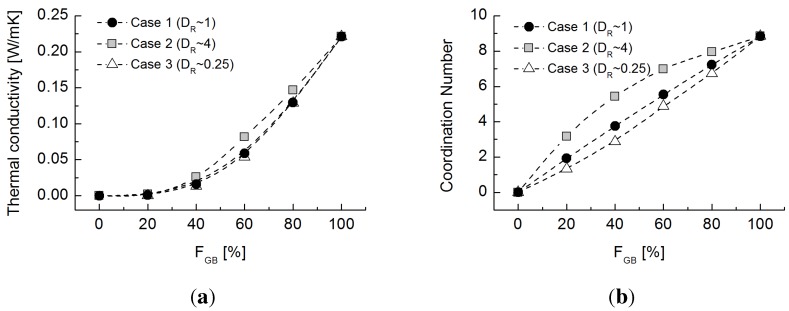
(**a**) Estimated thermal conductivity for Cases 1 to 3 by assuming that rubber particles are completely adiabatic (e.g., $k_{R}$ = 0 W/mK); (**b**) coordination number among glass bead particles for Cases 1 to 3.

Assuming that the rubber particles are completely adiabatic (e.g., $k_{R}=0$ W/mK), the estimated thermal conductivity values show a non-linear evolution, as presented in Figure 7a. In this simulation, the role of the rubber particles is ignored in considering thermal transfer. Hence, thermal conductivities are determined solely by the positions of and connections between the glass beads. Cases 1 and 3 show similar trends to that of the experimental results, whereas the convex evolution in the experiments for Case 2 is characterized by a concave evolution in the simulation. This change is attributed to the significant effect of the large, less conductive rubber particles. The coordination numbers for glass beads (Figure 7b) within the range of the effective zone are highest in Case 2 and lowest in Case 3. The sequence of the coordination number for the three cases is similar to that for the thermal conductivity estimation (Case 2 > Case 1 > Case 3). Although the difference in coordination number is relatively large between the different cases, the estimated thermal conductivity values are similar. Combining these observations suggests that highly conductive particles provide the major heat conduction paths with a minor influence of the spatial configuration of the particles, but the less conductive particles, especially relatively large ones, still significantly affect conduction. The major paths of thermal conduction are not only within the particles, but also between them through their contacts. Therefore, the effect of the less heat-resistant particles prevails when such particles are relatively large, for a given volume of rubber particles. The network model is able to capture both the particle size and contact resistance effects to evaluate the thermal conductance map.

## 5. Discussion

### 5.1. Sand-Rubber Mixture Test

**Figure 8:**
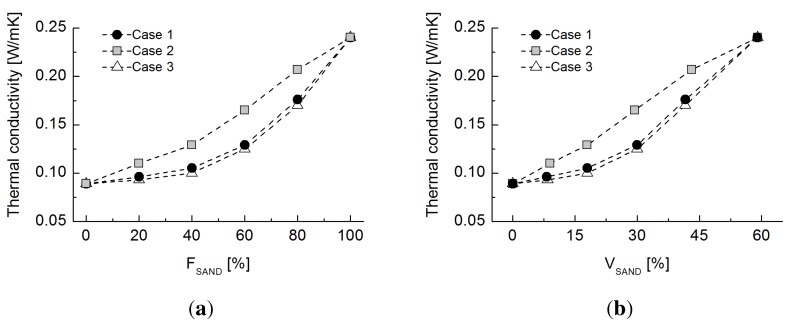
(**a**) Evolution of thermal conductivity values with varying volumetric fractions of sand for Cases 1 to 3; (**b**) density values with varying volumetric fractions of sand for Cases 1 to 3.

Glass beads were used to investigate the influence of particle size ratio on thermal conductivity and, despite their spherical shapes, to simplify the comparative numerical simulation. Hence, a sand-rubber mixture, a widely-studied mixture with possible usefulness deriving from the manipulation of its properties [4], was tested under similar experimental conditions (e.g., the same size ratio). Jumumjin sand ($D_{50}$ = 0.5 mm, $C_{u}$ = 1.16) was used to replace the glass beads, and the mixing conditions are summarized in Table 4. The porosity values were similar to those of the glass bead-rubber mixture specimens. Figure 8 shows the evolution of thermal conductivity with the varying volumetric fraction of sand. Although the tested sand is mainly comprised of quartz, whose thermal conductivity ranges from 3 to 8 W/mK and is higher than that of the glass bead raw material, the maximum thermal conductivity under dry conditions is similar to the experimental results shown in Figure 4. Case 2, which has the largest rubber particles, shows a quasi-linear trend with increasing sand fraction, whereas the behaviors of Cases 1 and 3 are similar to those in the glass bead-rubber mixture experiments. Despite the different particle shape, the particle size ratio still has the predominant effect on thermal conduction in natural sands.

**Table 4:** Volumetric fraction and porosity of sand-rubber mixture.

Vol.Fraction, $F_{S}$ (%)	Porosity		
	Case 1	Case 2	Case 3
0	0.62	0.61	0.62
20	0.59	0.55	0.58
40	0.55	0.55	0.55
60	0.50	0.51	0.50
80	0.48	0.46	0.49
100	0.41	0.41	0.41

### 5.2. Effect of Particle Contact

Heat transfer occurs mainly at direct contacts between conductive particles. To further investigate the effect of contact conduction, the electrical conduction of mixtures was tested. Electrical current flows only through particles that are in direct contact. The electrical current results therefore corroborate the significance of particle contact conditions determined by mixture fraction, with the effect of less conductive particles being eliminated. This corresponds to the case when the cutoff range parameter is not applicable (*i.e.*, hard-core percolation).

**Figure 9:**
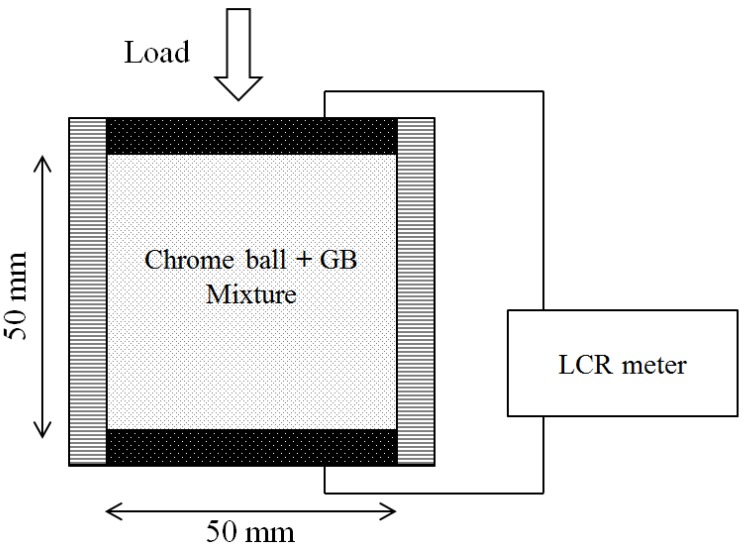
Experimental configuration for measuring the electrical conductivity of granular mixtures. The electrical conductance is obtained with two-point electrodes at the top and bottom plates.

Chrome balls were selected as electrically-conductive particles, and glass beads were used as insulators in these additional tests, in contrast to their conductive role in the thermal conductivity tests [17]. The balls and beads were mixed at 20% volumetric fraction increments. A vertical pressure of 112 kPa was applied to each mixture to ensure that the particles were in contact. The top and bottom stainless steel plates served as two-point electrodes. Subsequent measurement of the potential decrease between the two electrodes using an LCRmeter allowed the electrical resistance to be derived (Figure 9). The particle size ratios and the volumetric fractions of the tested mixtures are summarized in Table 5 and Table 6.

**Table 5:** Cases in chrome ball-glass bead mixture experimentation.

Case	Particle Diameter (mm)		$R_{d}$
	Chrome Ball	Glass Bead	
Case 1	1∼0.84	1.19∼0.3	∼1
Case 2	1∼1.68	2.38∼0.3	∼2
Case 3	1∼0.4	0.6∼0.3	∼0.5

**Figure 10:**
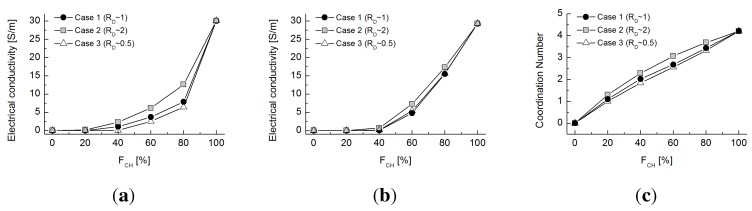
(**a**) Electrical conductivity values measured by two-point electrodes; (**b**) electrical conductivity values estimated by the network model; (**c**) coordination number estimated for virtual specimens with varying volumetric fractions of chrome balls for Cases 1 to 3.

Figure 10 shows the experimentally-measured electrical conductivity values, the values estimated using the network model and the coordination number for each of the three tested cases. Similar to the thermal studies, electrical conductivity values at $F_{CH}$ = 0 (0 W/mK) and 100% (32 W/mK) were first matched, and then, a curvature parameter *χ* = 0.016 was determined to capture the experimental trend. The electrical conduction occurs only via the chrome balls. As observed in the thermal conduction study, Case 2 (with glass beads larger than chrome balls) has a higher electrical conductivity than Cases 1 and 3. However, the difference is smaller than that in the thermal tests. This seems to be due to the size ratio in the electrical experiment being smaller than that in the thermal test. Furthermore, the insulating materials (*i.e.*, the glass beads) do not participate in conduction. Hence, the results are highly analogous to the thermal estimation, assuming that the rubber particles are completely adiabatic, and are similar to the results displayed in Figure 7a.

**Table 6:** Volumetric fraction and porosity of chrome ball-glass bead mixture.

Vol. Fraction, $F_{\mathrm{CH}}$ (%)	Porosity		
	Case 1	Case 2	Case 3
0	0.36	0.36	0.36
20	0.36	0.36	0.36
40	0.38	0.36	0.37
60	0.37	0.39	0.38
80	0.39	0.38	0.38
100	0.39	0.39	0.39

**Figure 11:**
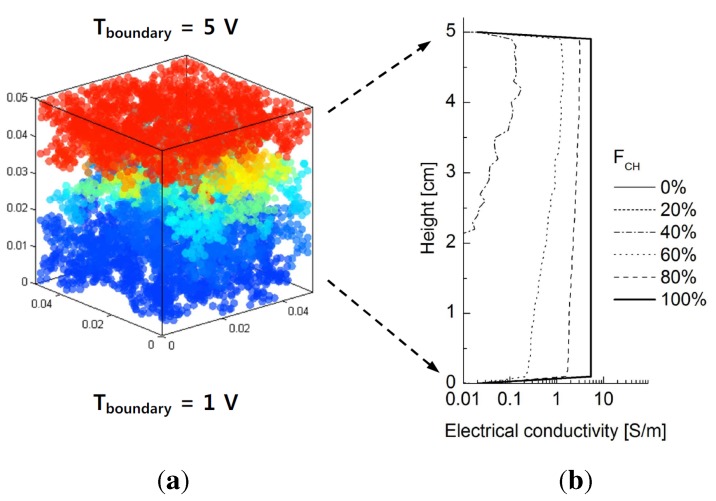
(**a**) Potential distribution ofthe chrome balls with respect to the height for Case 1: $F_{CH}$ = 60% at the steady-state condition. (**b**) Distribution of estimated electrical conductivity with respect to the height for different fractions of chrome balls.

Figure 11a shows the potential distribution of chrome ball particles for Case 1 at $F_{CH}$ = 60% at steady state. The electrical conductivity computed across the given cross-sectional areas with respect to height for different values of $F_{CH}$ are plotted in Figure 11b. Unlike thermal conduction, electrical conduction occurs only when the chrome balls are connected from top to bottom. Therefore, little variation in the electrical conductivity profiles with height is observed, unlike in Figure 6b, because chrome ball-chrome ball contacts are the only contributors to conduction. Furthermore, electrical conductivity is not measured below $F_{CH}∼40\%$, because there is not a continuously connected network of chrome balls connecting top to bottom. Combining the observations in Figure 10 and Figure 11 suggests that a fully developed contact networks of chrome balls emerges between $F_{CH}$ values of 20% to 40%.

**Figure 12:**
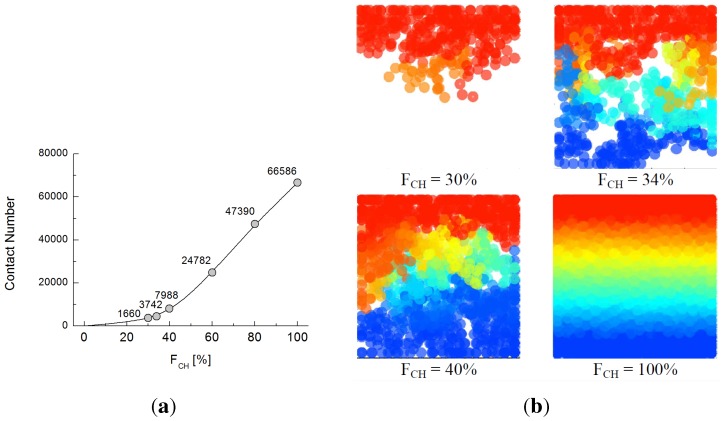
(**a**) Contact number along the volumetric fraction at Case 1; (**b**) connected chrome particles from the top to bottom boundary at different volume fractions for Case 1.

To discover the volume fraction that permits complete contact between the two electrodes, similar electrical conductivity tests were conducted at each one-percentage-point interval between $F_{CH}$ = 20% and 40% by using DEM and the network model. Figure 12a,b shows the increasing connected contact number of chrome balls from top to bottom and the 3D configuration of potentials of connected chrome particles at different volume fractions. A fully-developed connection is achieved when $F_{CH}$ exceeds 31%. The contact number of particles sharply increases with the increasing volumetric fraction of chrome balls, and the trend in the contact number of particles is similar to the trend in electrical conductivity. This shows that the contact number of conductive particles is the major factor determining conductivity.

## 6. Conclusions

This study experimentally investigated the effect of particle size ratio on the thermal conductivity of mixtures of highly conductive and less conductive materials. The experimental results of effective thermal conductivity and the thermal network model analysis emphasize that the relative size ratio of the granular materials that comprise the mixture is the dominant factor affecting conduction: it has a greater effect than does the volumetric fraction of each constituent. The observations lead to the following conclusions. The thermal conductivity of a granular mixture decreases with an increasing fraction of less conductive rubber particles. The relative particle size of the less conductive material determines the unique evolution of thermal conduction through its effect on the spatial configuration of the interconnectivity of the conductive particles. Therefore, the inclusion of large insulating materials favors thermal conduction to a greater extent than does the presence of small insulating particles, despite the mixtures having the same volumetric fractions of each component. This phenomenon enables both the specific weight and the thermal conductivity of a bulk material to be specifically designed by controlling both the volume fraction and the particle size of the materials in the mixture. The inter-particle coordination number is also influential factor determining thermal conductivity, and the modulation of the particle size ratio can, in turn, affect the coordination number for a given volumetric fraction. An additional test using natural sand corroborates the significance of the effect of particle size ratio over that of particle shape. The size effect becomes less dominant for a mixture containing completely insulating particles, as demonstrated in the electrical conduction test and the test using adiabatic rubber.

## References

[1] Balunaini U., Yoon S., Prezzi M., Salgado R. (2014). Pullout response of uniaxial geogrid in tire shred–sand mixtures. Geotech. Geol. Eng..

[2] Feng Z., Sutter K. (2000). Dynamic properties of granulated rubber/sand mixtures. ASTM Geotech. Test. J..

[3] Lee J., Dodds J., Santamarina J. (2007). Behavior of rigid-soft particle mixtures. J. Mater. Civil Eng..

[4] Lee J. (2012). Thermal and Electrical Behaviors of Selected Geomaterials. Ph.D. Thesis.

[5] Lee C., Shin H., Lee J. (2014). Behavior of sand–rubber particle mixtures: Experimental observations and numerical simulations. Int. J. Numer. Anal. Methods Geomech..

[6] Zornberg J., Cabral A., Viratjandr C. (2004). Behaviour of tire shred sand mixtures. Can. Geotech. J..

[7] Hamilton R., Crosser O. (1962). Thermal conductivity of heterogeneous two-component systems. Ind. Eng. Chem. Fundam..

[8] Yun T., Santamarina J. (2008). Fundamental study of thermal conduction in dry soils. Granul. Matter.

[9] Gustafsson S. (1991). Transient plane source techniques for thermal conductivity and thermal diffusivity measurements of solid materials. Rev. Sci. Instrum..

[10] Evans M., Lee J., Yun T., Valdes R. Thermal conductivity in granular mixtures: Experimental and numerical studies. Proceedings of the International Symposium on Deformation Characteristics of Geomaterials.

[11] Kim D., Kim Y., Lee J., Yun T. (2011). Thermal and electrical response of unsaturated hydrophilic and hydrophobic granular materials. ASTM Geotech. Test. J..

[12] Yun T., Evans T. (2010). Three-dimensional random network model for thermal conductivity in particulate materials. Comput. Geotech..

[13] Holtzman R., Silin D., Patzek T. (2010). Frictional granular mechanics: A variational approach. Int. J. Numer. Methods Eng..

[14] Choo J., Kim Y., Lee J., Yun T., Lee J., Kim Y. (2013). Stress-induced evolution of anisotropic thermal conductivity of dry granular materials. Acta Geotech..

[15] O’Sullivan C., Bray J. (2004). Selecting a suitable time step for discrete element simulations that use the central difference time integration scheme. Eng. Comput..

[16] Batchelor G., O’Brien R. (1977). Thermal or electrical conduction through a granular material. Proc. R. Soc. Lond. A Math. Phys. Sci..

[17] Lee J., Yun T. Electrical Conduction of Granular Media: Experimental and Numerical Studies. Proceedings of the GeoCongress 2012 State of the Art and Practice in Geotechnical Engineering.

